# Continuing versus withholding angiotensin receptor blocker (ARB)/calcium channel blocker (CCB) combination tablets during perioperative periods in patients undergoing minor surgery: a single-blinded randomized controlled trial

**DOI:** 10.1007/s00540-022-03053-8

**Published:** 2022-03-05

**Authors:** Kazuyo Takeuchi, Masakazu Hayashida, Osamu Kudoh, Naoko Niimi, Kumi Kataoka, Maho Kakemizu-Watanabe, Makiko Yamamoto, Atsuko Hara, Izumi Kawagoe, Keisuke Yamaguchi

**Affiliations:** Department of Anesthesiology and Pain Medicine, Juntendo University Graduate School of Medicine, 2-1-1 Hongo, Bunkyo-ku, Tokyo, 113-8421 Japan

**Keywords:** Angiotensin receptor blocker (ARB), Calcium channel blocker (CCB), Combination tablet, Hypotension, Renal function

## Abstract

**Purpose:**

This trial was conducted to compare effects of continuing versus withholding single-pill combination tablets consisting of angiotensin receptor blockers (ARBs) and calcium channel blockers (CCBs) on perioperative hemodynamics and clinical outcomes.

**Methods:**

Patients undergoing minor abdominal or urological surgery (*n* = 106) were randomly assigned to Group C, in which ARB/CCB combination tablets were continued until surgery, or Group W, in which they were withheld within 24 h of surgery. Perioperative hemodynamics and clinical outcomes were compared between the Groups.

**Results:**

The incidence of hypotension during anesthesia requiring repeated treatment with vasoconstrictors was higher in Group C than Group W (*p* = 0.0052). Blood pressure during anesthesia was generally lower in Group C than Group W (*p* < 0.05) despite significantly more doses of ephedrine and phenylephrine administrated in Group C (*p* = 0.0246 and *p* = 0.0327, respectively). The incidence of postoperative hypertension did not differ between Groups (*p* = 0.3793). Estimated glomerular filtration rate (eGFR) on the preoperative day did not differ between Groups (*p* = 0.7045), while eGFR was slightly lower in Group C than Group W on the first and third postoperative days (*p* = 0.0400 and *p* = 0.0088, respectively), although clinically relevant acute kidney injury did not develop.

**Conclusions:**

Continuing ARB/CCB combination tablets preoperatively in patients undergoing minor surgery increased the incidence of hypotension during anesthesia, increased requirements of vasoconstrictors to treat hypotension, and might deteriorate postoperative renal function, albeit slightly. These results suggest that withholding ARB/CCB tablets preoperatively is preferable to continuing them.

**Clinical trial registration:**

This trial is registered with the Japan Registry of Clinical Trials (jRCT) at Japanese Ministry of Health, Labour, and Welfare (Trial ID: jRCT1031190027).

## Introduction

Hypertension is a common condition affecting a significant percentage of populations. Current guidelines for the management of hypertension recommend a target blood pressure (BP) of < 140/90 or < 130/80 mmHg [1, 2]. Although first-line treatments for primary hypertension include calcium channel blockers (CCBs), angiotensin II receptor blockers (ARBs) or angiotensin-converting enzyme inhibitors (ACEIs), and diuretics, many hypertensive patients require combination therapy incorporating two or more antihypertensive agents to reach their target BP [1, 2]. In the practice of combination therapy, ARB/CCB single-pill, fixed-dose combination therapy, which can improve medication adherence, is frequently used as a rational treatment option [3, 4].

In patients with hypertension undergoing elective surgery, it is considered reasonable to continue medical therapy for hypertension until surgery [2]. Especially regarding CCBs, it has been considered reasonable to continue them until surgery [5, 6], as CCBs do not induce exaggerated hypotension during anesthesia [7, 8], and CCBs may improve patients’ postoperative outcomes [9, 10]. Regarding ACEIs and ARBs, the 2014 American Heart Association (AHA) guideline recommends that continuation of ACEIs and ARBs in the immediate preoperative period is reasonable [11], whereas the 2014 European Society of Cardiology (ESC) guidelines recommends transient discontinuation of ACEIs and ARBs before surgery [12], mainly based on the identical data showing that their continuation does not worsen patients’ outcomes, although it increases the risk of intraoperative hypotension [13, 14], as also confirmed by a later meta-analysis [15]. After publication of a large-scale observational cohort study showing that continuing ACEIs and ARBs perioperatively may worsen clinical outcomes in patients undergoing non-cardiac surgery [16], recent reviews and guidelines tend to recommend withholding ACEIs/ARBs [6, 17], although it may be required to restart ACEIs/ARBs after surgery as soon as possible because delayed or omitted reinstitution may worsen clinical outcomes [6, 18, 19].

As mentioned above, CCBs are generally continued throughout the perioperative period while ARBs recently tend to be withheld preoperatively. However, it remains to be known whether ARB/CCB combination tablets should be continued or withheld preoperatively. The present study was conducted to compare effects of continuing versus withholding ARB/CCB combination tablets preoperatively on perioperative hemodynamics and clinical outcomes.

## Methods

The present single-blinded randomized controlled trial (RCT) was approved by the Institutional Review Board (IRB) of Juntendo University Hospital (JUH) and Juntendo Tokyo Koto Geriatric Medical Center (KGMC) (common approval number: J18-019), and registered with the Japan Registry of Clinical Trials (jRCT) at Japanese Ministry of Health, Labour, and Welfare (Trial ID: jRCT1031190027). Written informed consent was obtained from each patient.

### Patients

Enrolled were ASA-PS II patients aged 20–80 years with primary hypertension treated with ARB/CCB combination tablets, who were scheduled to undergo minor abdominal surgery or minor urological surgery under general anesthesia between May, 2019 and March, 2020. Minor surgery was arbitrarily defined as surgery of which scheduled surgical time was 3 h or shorter and in which significant perioperative bleeding was unexpected. Excluded were patients with severe systemic disease corresponding to ASA-PS III or more, and patients with severe renal insufficiency defined as estimated glomerular filtration rate (eGFR) less than 30 mL/min/1.73 m^2^.

### ARB/CCB combination tablets

ARB/CCB combination tablets under evaluation included 12 originator products—EXFORGE^®^ Combination Tablets (valsartan/amlodipine, 80 mg/5 mg) (Novartis Pharma K.K., Tokyo, Japan), REZALTAS^®^ Combination Tablets LD and HD (olmesartan/azelnidipine, 10 mg/8 mg and 20 mg/16 mg) (Daiichi Sankyo Co., Ltd., Tokyo), UNISIA^®^ Combination Tablets LD and HD (candesartan/amlodipine, 8 mg/2.5 mg and 8 mg/5 mg) (Teva Takeda Yakuhin Ltd., Osaka, Japan), Micamlo^®^ Combination Tablets AP and BP (telmisartan/amlodipine, 40 mg/5 mg and 80 mg/5 mg) (Nippon Boehringer Ingelheim Co., Ltd., Tokyo), AIMIX^®^ LD and HD (irbesartan/amlodipine, 100 mg/5 mg and 100 mg/10 mg) (Sumitomo Dainippon Pharma Co., Ltd., Osaka), ZACRAS^®^ Combination Tablets LD and HD (azilsartan/amlodipine, 20 mg/2.5 mg and 20 mg/5 mg) (Takeda Pharmaceutical Co. Ltd., Tokyo), ATEDIO^®^ Combination Tab. (valsartan/cilnidipine, 80 mg/10 mg) (Mochida Pharmaceutical Co., Ltd., Tokyo), and generic products of these combination tablets. Consequently, these tablets included 12 different ARB/CCB combination tablets with different components and/or doses.

### Registration and randomization

Patients were registered in the order of patients’ consent. Patients were randomly assigned to either continuing drug group (Group C), in which ARB/CCB combination tablets were continued until the evening before surgery or the morning of surgery, or withholding drug group (Group W), in which ARB/CCB combination tablets were withheld within 24 h of surgery, using the envelope method applied separately to patients scheduled for abdominal surgery and those scheduled for urological surgery.

### Anesthesia management

Patients were allowed to eat freely on the day before surgery. They were allowed to drink clear liquids freely until 2 or 3 h before scheduled operation room entry time. Patients not allocated to the first case of the day received i.v. infusion of Ringer’s solution, starting on the morning.

Attending anesthesiologists were blinded to patients’ Groups. They were just instructed to anesthetize patients with general anesthesia alone, by inducing general anesthesia with opioids (fentanyl and/or remifentanil) and propofol and by maintaining anesthesia with inhalational anesthetics (sevoflurane or desflurane) and opioids. Otherwise, methods of the anesthesia management were left to the discretion of attending anesthesiologists depending on each patient’s condition and each surgical procedure, considering patient safety to be the first priority. At the end of surgery, i.v. fentanyl and/or i.v. acetaminophen were given for immediate postoperative analgesia. After anesthesia, patients were transferred from the operation room (OR) to the post-anesthesia care unit (PACU), and then to the ward.

In the OR and PACU, significant hypotension defined as systolic BP (SBP) < 80 mmHg was treated with i.v. injections of vasopressors—i.v. ephedrine (4 or 5 mg/bolus) or i.v. phenylephrine (0.1 mg/bolus). If required, continuous infusion of phenylephrine was used. Significant hypertension defined as SBP > 160 mmHg persisting for 15 min was treated with vasodilators—i.v. nicardipine (0.5 mg/bolus) and/or transdermal isosorbide dinitrate (40 mg). If required, continuous infusion of nicardipine was used.

### Perioperative monitoring of vital signs

Preoperatively in the ward, BP, percutaneous oxygen saturation (SpO_2_), a pulse rate (as a surrogate of the heart rate [HR]), a respiratory rate, and body temperature (BT) were measured and recorded on the medical record at least three times—after hospitalization, on the evening before surgery, and on the morning of surgery. In the OR, BP was measured and recorded on the electronical anesthesia record every 2.5 min. HR, SpO_2_, end-tidal carbon dioxide tension (ETCO_2_) and BT were monitored continuously and recorded every one minute. In the PACU, BP, BT, and pain scores and nausea scores rated on an 11-point numerical rating scale (NRS) were measured and recorded every 5 min. HR and SpO_2_ were monitored continuously and recorded every minute. Patients stayed in the PACU at least for 20 min until stable vital signs and adequate pain control were achieved. Postoperatively in the ward, the five above-mentioned vital signs and NRS pain and nausea scores were recorded every 2 h during the first 6 h, and thereafter every 6 h until the next morning. Afterwards, these variables were measured at least three times a day until discharge.

### Collection of data on perioperative BPs and HRs

BPs at the following 20 perioperative time points were noticed, including (1) BP after hospitalization, (2) BP on the morning of surgery before drug administration, (3) BP after entering the OR before anesthesia, (4) the lowest BP after induction of anesthesia and before tracheal intubation, (5) the highest BP after tracheal intubation, (6) the lowest BP before surgery, (7) BP at the start of surgery, (8) the highest BP during surgery, (9) the lowest BP during surgery, (10) BP at the end of surgery, (11) the highest BP after extubation, (12) BP at the end of anesthesia, (13) BP after entering the PACU, (14) the highest BP in the PACU, (15) the lowest BP in the PACU, (16) BP at the end of PACU stay, (17) BP after return to the ward, (18) the highest BP in the ward, (19) the lowest BP in the ward, and (20) BP on the morning of the first postoperative day before drug administration. Likewise, HRs at the 20 perioperative time points were noticed.

### Blood tests

Routine blood tests were performed at least three times—on the day before surgery (PreOD), and on the first and third postoperative days (POD 1 and POD 3). Thereafter, they were performed repeatedly, as required.

### Study endpoints

The primary endpoint was to investigate the effect of continuing versus withholding ARB/CCB combination tablets on the incidence of significant hypotension during anesthesia, defined as SBP < 80 mmHg requiring treatment with vasoconstrictors. Secondary endpoints included effects of continuing versus withholding drugs on (1) perioperative BPs and HRs, (2) vasoactive agents required during the perioperative period, and (3) clinical outcomes, such as postoperative complications, including abnormal blood test data, and postoperative hospital stay.

### Statistical analysis

Many RCTs investing the effect of continuing versus withdrawing ARBs or ACEIs, cited in a recent meta-analysis [15], were conducted by enrolling 100 patients or less to detect a significant difference in the incidence of intraoperative hypotension. Therefore, we aimed to enroll 110 patients (100 at the JUH and 10 at the KGMC).

Continuous data are presented as Mean ± SD (Range) or Median (Interquartile range) (10th percentile–90th percentile or Range), according to data types indicated by the Kolmogorov–Smirnov test. Categorical data are presented as Number (%). Parametric data were compared between Groups with the unpaired *t* test and within each Group with the paired *t* test or repeated measures ANOVA followed by the Scheffe test. Nonparametric data were compared between Groups with the Mann–Whitney *U* test and within each Group with the Friedman test and the Wilcoxon signed-rank test. Categorical data were compared between Groups with the Chi-square test. Since BPs and HRs in the ward and PACU were mostly normally distributed, although occasionally, they were non-normally distributed in the OR, they were treated as parametric data. Conversely, most blood tests data were non-normally distributed, and they were treated as nonparametric data. However, most of percent changes in blood laboratory tests data investigated were normally distributed, they were treated as parametric data. Statistical analysis was performed with SPSS 25.0 (SPSS, Chicago, IL, USA) and StatFlex ver. 7 (ARTECH, Osaka, Japan). A *p* value < 0.05 was considered statistically significant.

## Results

### Clinical backgrounds

A hundred and ten patients were registered. However, four patients (one in Group C and three in Group W) were excluded because the exclusion criterion was met by two patients, and the method of anesthesia was changed from general anesthesia alone to other anesthesia methods in two patients. As a result, a total of 106 patients (54 in Group C and 52 in Group W) were investigated.

Patients were treated with any of 12 different ARB/CCB combination tablets, kinds of which did not differ between Group C and Group W (*p* = 0.5738 by the Chi-square test). Further, there were no significant differences between the Groups in patients’ demographic, surgical, and anesthetic characteristics (except for requirements of vasoconstrictors), albeit on the condition that amounts of bleeding and urine during transurethral procedures were assumed to be zero (Table 1). None of patients required blood transfusion perioperatively.

**Table 1:** Data on patients’ demography, surgery, anesthesia, and postoperative course

Variables	Group C (*n* = 54)	Group W (*n* = 52)	*p* values
Age (years)	69.4 ± 8.3 (47–80)	68.3 ± 9.1 (46–80)	0.5236
Sex, males	50 (92.6%)	46 (88.5%)	0.4670
Sex, females	4 (7.4%)	6 (11.5%)	
Body height (cm)	165.6 ± 8.2 (148–186)	166.0 ± 7.2 (150–183)	0.7670
Body weight (kg)	70.63 ± 70.63 (47.2–97.1)	68.22 ± 11.53 (48.8–94.7)	0.3151
Surgical procedures			
Robot-assisted laparoscopic prostatectomy	12 (22.2%)	14 (26.9%)	0.5831
Transurethral resection of bladder tumor	13 (24.1%)	7 (13.5%)	
Laparoscopic inguinal hernia repair	11 (20.4%)	7 (13.5%)	
Transurethral or percutaneous lithotripsy	6 (11.1%)	9 (17.3%)	
Laparoscopic gastroenterological surgery	6 (11.1%)	8 (15.4%)	
Laparoscopic cholecystectomy	6 (11.1%)	7 (13.5%)	
Surgical time (min)	107 (60, 155) (16–204)	107 (73.5, 146) (15–241)	0.9119
Anesthesia management			
Anesthesia time (min)	149.5 (83, 215) (28–269)	156 (111.5, 200.5) (36–304)	0.8151
Airway management, tracheal intubation	49 (90.7%)	45 (86.5%)	0.4948
Airway management, supra-glottis device	5 (9.3%)	7 (13.5%)	
Infusion during anesthesia (mL)	800 (400, 1500) (150–2600)	700 (425, 925) (150–2805)	0.1690
Bleeding during anesthesia (mL)	3 (1, 15) (0–300)	5 (2.5, 25) (0–200)	0.2585
Urine output during anesthesia (mL)	30 (0, 100) (0–825)	32.5 (0, 200) (0–750)	0.7305
Propofol (mg) given for induction	100 (90, 140) (60–170)	100 (80, 120) (40–160)	0.1398
Inhalational anesthetics, sevoflurane (S)	16 (29.6%)	12 (23.1%)	0.4443
Inhalational anesthetics, desflurane (D)	38 (70.4%)	40 (76.9%)	
Concentration of S for maintenance (%)	1.5 (1.5, 2.0) (1.0–3.0) (*n* = 16)	1.75 (1.25, 2.1) (1.0–3.5) (*n* = 12)	0.9242
Concentration of D for maintenance (%)	4.0 (4.0, 4.25) (3.5–5.0) (*n* = 38)	4.0 (4.0, 4.4) (3.8–8.0) (*n* = 40)	0.8878
Fentanyl given during anesthesia (μg)	200 (100, 300) (50–570)	200 (100, 300) (100–550)	0.3668
Remifentanil given during anesthesia (μg)	120 (50, 220) (0–4.2)	150 (100, 250) (0–7.3)	0.1517
Intravenous acetaminophen (mg)	1000 (0, 1000) (0–1000)	1000 (375, 1000) (0–1000)	0.2358
Ephedrine given during anesthesia (mg)	10 (4, 25) (0–40)	5 (0, 15) (0–35)	0.0246
Phenylephrine given during anesthesia (mg)	0 (0, 0.3) (0–3.5)	0 (0, 0) (0–1.0)	0.0327
Patients requiring vasoconstrictors (VCs)^a^	44 (81.5%)	39 (75.0%)	0.4183
Patients requiring VCs more than once	42 (77.8%)	27 (51.9%)	0.0052
Postoperative course			
Patients requiring vasodilators^b^	7 (13.0%)	10 (19.2%)	0.3793
Postoperative complications^c^	0 (0%)	2 (3.8%)	0.1457
Postoperative hospital stay (days)	4 (3, 9) (3–11)	4 (3, 7) (3–22)	0.7592
Postoperative hospital stay ≥ 14 days	0 (0%)	2 (3.8%)	0.1457

Data are shown as Mean ± SD (Range), Median (25th and 75th percentiles) (Range), or Number (%), and were analyzed with the unpaired *t* test, Mann–Whitney *U* test, or Chi-square test, as appropriate

^a^Vasoconstrictors (VCs) included ephedrine and phenylephrine

^b^Vasodilators included nicardipine, isosorbide dinitrate, and both drugs in one, four, and two patients, respectively, in Group C (*n* = 7), and in five, two, and three patients, respectively, in Group W (*n* = 10)

^c^Postoperative complications developed in two patients in Group W, including functional ileus and surgical site infection

### Incidences of intraoperative hypotension and postoperative hypertension

The number of patients who developed significant hypotension during anesthesia, defined as SBP < 80 mmHg requiring treatment with ephedrine and/or phenylephrine at least once, did not differ between Group C and Group W (Table 1). However, the number of hypotensive patients requiring repeated treatment with these vasoconstrictors more than once was greater in Group C than in Group W (Table 1). Doses of ephedrine and phenylephrine required to treat hypotension were higher in Group C than in Group W (Table 1). During anesthesia, none of patients required vasodilators to treat hypertension. After anesthesia, none of patients required vasoconstrictors to treat hypotension, whereas 17 patients required vasodilators to treat postoperative hypertension. The number of patients requiring vasodilators did not differ between the Groups (Table 1).

### Hemodynamic changes during the perioperative period

**Fig. 1:**
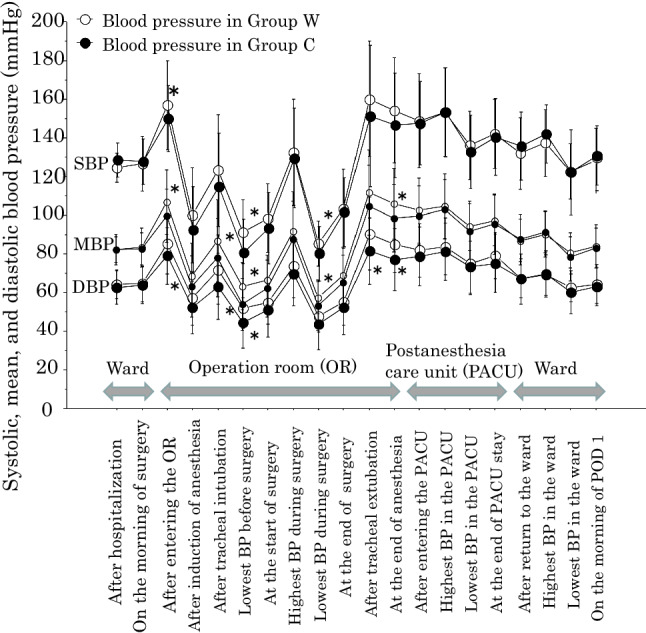
Perioperative changes in systolic blood pressure (SBP), mean blood pressure (MBP), and diastolic blood pressure (DBP) in Group C and Group W. Closed circles indicate Group C and open circles indicate Group W. See detailed explanations for 20 perioperative time points in the methods section in the text. *p < 0.05 between Group C and Group W by the unpaired *t* test.

Systolic, mean, and diastolic BP (SBP/MBP/DBP) changed significantly in both Groups during the perioperative period (p < 0.0001 for each by the ANOVA). Briefly, BP after entering the OR before anesthesia was higher; BPs during anesthesia were lower (except for the highest BP after intubation and the highest BP during surgery); BPs after extubation in the OR and PACU were higher; compared with BP on the morning of surgery in both Groups (Fig. 1). For example, mean values of SBP/DBP increased from 126.6/70.7 mmHg on the morning of surgery to 156.9/91.7 mmHg after entering the OR in Group W (*p* < 0.0001 by the Scheffe test), while in Group C, the mean values increased from 126.4/70.1 mmHg to 148.4/85.3 mmHg (*p* < 0.0001). These mean values in the OR were lower in Group C than in Group W, as described below (Fig. 1). Postoperative BPs in the ward were not different from BP on the morning of surgery in both Groups (Fig. 1).

Preoperatively in the ward and postoperatively in the PACU and ward, BPs did not differ between the Groups (Fig. 1). In the OR, however, BPs measured at several time points were lower in Group C than in Group W (*p* < 0.05 by the unpaired *t* test), including SBP/MBP/DBP after entering the OR before anesthesia, the highest MBP/DBP after tracheal intubation, the lowest SBP/MBP/DBP after tracheal intubation before surgery, the lowest intraoperative SBP/MBP, the highest DBP after extubation, and MBP/DBP at the end of anesthesia (Fig. 1).

HR changed significantly during the perioperative period in both the Groups (*p* < 0.0001 for each by the ANOVA). Perioperative HRs mostly did not differ between the Groups, except for HR at the start of surgery and the lowest HR during surgery, which were higher in Group C than in Group W (*p* < 0.05 for each by the unpaired *t* test).

### Changes in blood tests data

**Fig. 2:**
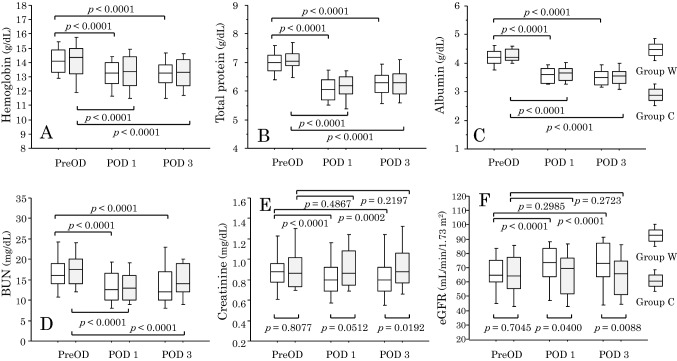
Blood concentrations of **A** hemoglobin, **B** total protein, **C** albumin, **D** blood urea nitrogen (BUN), **E** creatinine, and **F** estimated glomerular filtration rate (eGFR) measured on the preoperative day (PreOD), the first postoperative day 1 (POD 1), and the third postoperative day (POD 3). Data are expressed as box and whisker plots. A solid line in the box depicts the median. Ends of the box represent the 75th and 25th percentiles. Whiskers represent the 90th and 10th percentiles. White and gray boxes represent Group W and Group C, respectively. Intra-group comparisons were performed with the Wilcoxon signed-rank test, and inter-group comparisons were performed with the Mann–Whitney *U* test.

There were no inter-group differences in blood tests data measured on the preoperative day (PreOD), including hemoglobin (Hb), total protein (TP), albumin, aspartate aminotransferase (AST), alanine aminotransferase (ALT), blood urea nitrogen (BUN), creatinine, and estimated glomerular filtration rate (eGFR) (*p* > 0.05 by the Mann–Whitney *U* test) (Fig. 2).

In the total cohort, these variables, other than AST and ALT, changed significantly (*p* < 0.05 for each by the Friedman test). Hb, TP, albumin, and BUN were lower on POD 1 and POD 3 than on PreOD (Fig. 2). Creatinine was lower on POD 1 and tended to be lower on POD 3 than on PreOD (*p* = 0.0002 and *p* = 0.0506, respectively, by the Wilcoxon test), while eGFR was higher on POD 1 and POD 3 than on PreOD (*p* < 0.0001 and *p* = 0.0133, respectively). In the total cohort, percent decreases in TP, albumin, and BUN from PreOD to POD 1 (12.8 ± 5.8%, 15.0 ± 5.7%, and 19.4 ± 24.2%, respectively) were more than a percent decrease in Hb (5.7 ± 5.4%, *p* < 0.0001 for each by the paired *t* test). Likewise, percent decreases in TP, albumin, and BUN from PreOD to POD 3 (10.6 ± 6.3%, 17.1 ± 6.5%, and 15.2 ± 24.2%, respectively) were more than a percent decrease in Hb (6.3 ± 6.4%, *p* < 0.0001 for each).

In both Groups, Hb, TP, albumin, and BUN were lower on POD 1 and POD 3 than on PreOD (Fig. 2), and these variables on each day were not different between the Groups (Fig. 2). In Group W, creatinine and eGFR changed significantly (*p* = 0.0006 for both by the Friedman test). Creatinine was lower while eGFR was higher on POD 1 and POD 3 than on PreOD (Fig. 2). In Group C, however, creatinine and eGFR did not change significantly (*p* > 0.05 for each by the Friedman and Wilcoxon tests) (Fig. 2). As a result, creatinine on POD 1 tended to be higher and creatinine on POD 3 was higher in Group C than in Group W, while eGFR on POD 1 and eGFR on POD 3 were lower in Group C than in Group W (Fig. 2). Follow-up blood tests performed on 28.3 ± 4.5 days after surgery in 42 patients in Group C and 43 patients in Group W revealed no differences between the Groups in creatinine and eGFR (*p* = 0.7583 and *p* = 0.7954, respectively, by the Mann–Whitney *U* test).

AST or ALT did not change significantly in Group C or Group W (p > 0.05 for each by the Friedman test), although AST and/or ALT increased to extremely high levels (> 100 IU/L) on POD 1 and/or POD 3 in two patients (one in Group W and one in Group C). AST or ALT on PreOD, POD 1 or POD 3 did not differ between the Groups (*p* > 0.05 for each by the Mann–Whitney *U* test).

### Other data associated with clinical outcomes

Other than above-mentioned changes in blood tests data, clinically relevant anesthesia-related adverse events did not develop, although surgery-related complications to prolong hospitalization developed in two patients in Group W (functional ileus and surgical site infection). Hospital stay after surgery was not different between the Groups (Table 1).

## Discussion

In the present study, the incidence of hypotension during anesthesia defined as SBP < 80 mmHg requiring treatment with vasoconstrictors at least once did not differ between the Groups. However, the incidence of hypotension requiring repeated treatment with vasoconstrictors was higher in Group C than in Group W. BP during anesthesia was mostly significantly lower in Group C despite significantly more doses of vasoconstrictors administrated in Group C. The incidence of postoperative hypertension treated with vasodilators did not differ between the Groups. Although eGFR was not different between the Groups preoperatively, eGFR was lower in Group C than in Group W postoperatively.

In both patients receiving and not receiving ARB/CCB combination tablets, BP after entering the OR before anesthesia significantly increased, compared with BP on the morning of surgery. BP after entering the OR was lower in patients receiving than not receiving ARB/CCB combination tablets. These results suggested that continuing ARB/CCB combination tablets helps to ameliorate hypertension in the OR before anesthesia. However, because it is unknown whether abnormal hypertension before surgery is associated with worse postoperative outcomes [6], clinical significance of the possible capacity of ARB/CCB combination tablets to ameliorate preoperative hypertension also seems unknown. During anesthesia and surgery, BPs at various time points were lower in patients receiving than not receiving ARB/CCB combination tablets. Although the incidence of hypotension requiring treatment with vasoconstrictors at least once did not differ between patients receiving and not receiving ARB/CCB combination tablets, the incidence of hypotension requiring repeated treatment with vasoconstrictors was higher, and doses of vasoconstrictors required to treat hypotension were higher in patients receiving than not receiving ARB/CCB combination tablets. Reportedly, intraoperative hypotension to certain degrees could be associated with increased risks of postoperative adverse outcomes, including acute kidney injury (AKI), myocardial injury, stroke, overall organ injury, and even mortality [16, 20]. Further, the incidence of postoperative hypertension requiring vasodilator therapy did not differ between both patients, indicating that continuing ARB/CCB combination tablets did not help to prevent postoperative hypertension. Therefore, we could not find an advantage of continuing ARB/CCB combination tablets, although we could not find a critical disadvantage of this practice either, since clinically relevant adverse outcomes associated with intraoperative hypotension developed in none of our patients.

Clinically relevant AKI according to its definition [21] developed in none of our patients. Rather, postoperative renal function apparently improved because postoperatively, creatinine significantly decreased and eGFR significantly increased in our total cohort. However, such an apparent improvement in postoperative renal function might result from decreased creatinine due to postoperative hypercatabolism from fasting and/or surgical stress, which results in breakdown of proteins including muscles [22, 23]. Our data showing that total protein, albumin, and BUN (a product of protein metabolism) decreased significantly after surgery support this assumption. Although decreases in concentrations of proteins, BUN, and creatinine might also result from hemodilution due to perioperative blood loss and/or water retention following surgery [24], much greater decreases in total protein, albumin, and BUN compared to a decrease in hemoglobin suggest that such decreases primarily resulted from postoperative hypercatabolism, rather than hemodilution. eGFR is accepted as a more accurate estimate of renal function, compared with serum creatinine, because eGFR compensates for age- and sex- associated decreases in creatinine due to reduced muscle mass [25]. It should be noted, however, that in critically ill patients, even eGFR can overestimate renal function [26]. Our data suggest that postoperative renal function can apparently improve even after minor surgery possibly because eGFR might overestimate renal function even in such situations.

Therefore, creatinine might decrease normally and eGFR might increase normally postoperatively, thereby resulting in apparent improvement in postoperative renal function. In our patients, however, such changes were observed only in patients not receiving ARB/CCB combination tablets, and not in those receiving these drugs. As a result of apparently unchanged postoperative renal function in patients receiving these drugs in contrast to apparently improved renal function in patients not receiving them, creatinine was higher or tended to be higher and eGFR was lower postoperatively in patients receiving than not receiving ARB/CCB combination tablets, despite that both patients had comparable renal function preoperatively. Such small inter-group differences in indicators of renal function that appeared postoperatively might indicate that clinically latent, but not clinically relevant, deterioration of renal function developed postoperatively in patients receiving ARB/CCB combination tablets, relative to those not receiving these drugs. While ARBs and CCBs exert renoprotective effects in patients with hypertension and chronic kidney disease [27], intraoperative hypotension might increase the risk of postoperative adverse outcomes, including AKI [16, 20]. In our patients, more significant intraoperative hypotension in patients receiving ARB/CCB combination tablets might result in clinically latent deterioration of renal function even after minor surgery possibly because effects of significant intraoperative hypotension exaggerated by ARBs/CCBs outweighed their renoprotective effects. These results suggest that withholding ARB/CCB combination tablets is a more acceptable clinical practice than continuing these drugs.

There are some limitations of the study. First, we did not follow-up all the patients after discharge especially regarding their renal function primarily because we had not expected a possible unfavorable effect of continuing ARB/CCB combination tablets on renal function until we analyzed data. However, there were no patients who developed evident AKI as far as we searched. Second, our study evaluated effects of 12 different ARB/CCB combination tablets with different components and/or doses and thus with different vasodilatory potencies because it was quite difficult to recruit a sufficient number of patients receiving a single ARB/CCB combination tablet. Therefore, it may not be practical to standardize medication protocols of ARB/CCB combination tablets based on our results. Third, the sample size might be too small to perform multiple comparisons and to analyze multiple endpoints. Therefore, we could not obtain a definite conclusion from our data except for that intraoperative hypotension requiring repeated treatment with vasoconstrictors occur more frequently when ARB/CCB combination tablets are continued than withheld. However, we do not intend to conduct a further large-scale study to verify the present results especially in patients undergoing major surgery because from our results, the possibility can not be excluded that continuing ARB/CCB combination tablets perioperatively may cause AKI in some sensitive patients.

In conclusion, continuing ARB/CCB combination tablets preoperatively in patients undergoing minor surgery increased the incidence of hypotension requiring repeated treatment with vasoconstrictors during anesthesia, increased requirements of vasoconstrictors to treat hypotension, and might deteriorate postoperative renal function, albeit only slightly. Further, continuing ARB/CCB combination tablets preoperatively did not help to prevent postoperative hypertension. Therefore, withholding ARB/CCB combination tablets within 24 h of surgery seemed to be a more acceptable clinical practice than continuing these drugs until surgery.

## Data Availability

The datasets related to this study are available from the corresponding author on reasonable request.
